# Effects of a high fat diet on bone of growing rats. Correlations between visceral fat, adiponectin and bone mass density

**DOI:** 10.1186/1476-511X-7-16

**Published:** 2008-04-28

**Authors:** Gerard Lac, Helian Cavalie, Edmond Ebal, Odile Michaux

**Affiliations:** 1 Biologie des Activités Physiques et Sportives (BAPS), Labo Biologie B, Les Cézeaux, 63177 AUBIERE cedex, France

## Abstract

In this study, we investigated some bone parameters (bone mineral content, bone mineral density, skeleton area) in growing rats fed with a high fat diet. Correlations between bone and body composition parameters are reported. Two groups of Wistar male rats (35 days old, body mass 80 ± 6 g) were used. Water and food were given "*ad libitum*" during 10 weeks. Sixteen rats (L) were given a lipid enriched diet and were compared to 16 rats (S) fed with a standard diet. Body composition and bone parameters were assessed using DXA. Results indicated that L rats had lower body mass, lean body mass; fat mass was not different between the two groups. Bone mineral content, bone mineral density, skeleton area of L rats were lower compared with S rats. Significant correlations were noted between body composition, adiponectin and bone parameters. High fat diet intake during the growing period has deleterious effects on bone parameters in rats. This study confirms in growing rats that a high fat diet is pathogenic, including bone metabolism.

## Background

Bone mass components like mineral content, mineral density and skeleton area are influenced by genetic and environmental factors (ethnicity, age, gender, height, body mass, pubertal stage...) [1]. Diet composition, in particular fat content, has been shown to act negatively on bone status. Children treated with ketogenic diet (very high fat and low carbohydrate diet) showed a poor bone mineral status [2]. In animal models (rat), a western-style diet, i.e. high fat content, will result in low bone mass (lower bone mineral content) and poor bone quality [3]. Diets with high saturated fat content have an adverse effect on bone mineralization in growing animals [4]. Diets high in fat decrease whole bone mineralization [5]. Moreover, the quality of fat may also affect the skeleton characteristics; healthy term infants fed with a diet containing palm olein as the predominant oil in the fat blend, had significantly lower bone mineral content and bone mineral density than those fed with a diet containing any palm olein [6]. More surprisingly, weanling rats fed with a fish oil diet, showed any effects on bone mechanical properties in males and showed even negative effects in females [7]. Thus, it seems that the quality as well as the quantity of lipids in the diet may exert adverse effects on bone development. It is well known that, western style diet affects body composition: high percentage of fat mass, especially visceral fat. Some studies have investigated the possible relationship between body composition (particularly fat) and bone strength [8] and also the correlations between fat and one of its adipokine: adiponectin, with bone mineral density [9].

Studies on animals assessed biomechanical properties and ashes content of one (femur) or two (femur + vertebrae) bones. Studies using DXA were only made on humans.

In this study, we investigated in growing rats the effects of a high fat diet on whole body skeleton measured by DXA (bone mineral content, bone mineral density, skeleton area). Moreover we evaluated the possible correlations between bone and body composition parameters and also with the adipokine adiponectin.

## Materials and methods

### Animals and treatments

This experiment was done in accordance with current legislation on animal experiments in France and was agreed by the local committee of ethics.

Thirty two Wistar male rats (35 days old, body mass 80 ± 6 g) were investigated. Groups of four rats were housed in 22 × 22 × 18 cm plastic cages at 22 ± 1°C, with a 12: 12 hr light: dark cycle. Water and food were given "*ad libitum*" during 10 weeks.

Sixteen rats (L) were given a Lipid enriched diet made of a mixture of 75 g of laboratory chow (UAR A04, Villemoisson sur Orge, France) of known composition (72.2% of carbohydrates, 7.7% of fats, 20% of proteins calories and 0.9 g/100 g Ca) to which was added 15 grams of commercial vegetable oil (fats 100% comprising 12% of saturated fatty acids, 41% of monounsaturated fatty acids, 47% of polyunsaturated fatty acids) and 10 grams of powdered skimmed milk (lactose, 51.7%; fats, 1%; proteins, 36% and 1.3 g/100 g Ca); thus, the global caloric composition and total energy value of this experimental diet were 41.5% of carbohydrates, 38.5% of fats, 20% of protein and 425 Kcal/100 g and the Ca content was 0.8 g/100 g. The other 16 rats (S) received a Standard diet.

### Measurements

Animals were weighed daily for a permanent follow up of body mass (BM).

Food intake was assessed by differential weighing daily for each group of 4 rats.

Fat mass (FM), lean body mass (LBM), Bone mineral content (BMC), bone mineral density (BMD) and Skeleton area (SA) were assessed under chloral anaesthesia by dual energy X-ray absorptiometry (DXA) using a Hologic QDR 4500A (version 11.2.5) densitometer calibrated for small animals [10]. Visceral fat mass was assessed by weighing the left perirenal adipose fat pad. Adiponectin was assayed with a commercial ELISA kit (BioCat, Germany) following manufacturer recommendations.

### Statistical analysis

All data are reported means ± SD. Comparisons between groups were made using Student *t *test for unpaired series. Correlations were made by a simple regression analysis between variables; the level of significance was set at p < 0.05.

## Results

### Energy intake

**Figure 1:**
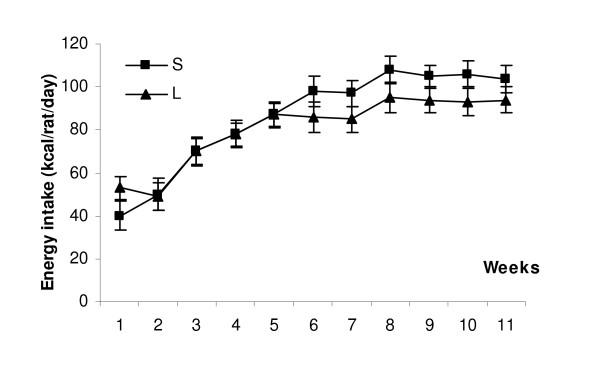
**Energy intake (Kcal/rat/day) in S and L rats.** Difference between S and L are not significant.

L rats adjusted their energy intake at the same level than S rats very quickly during the 5 first weeks. Although a decrease during the last weeks, resulting in a lower total energy intake in L than in S rats throughout the protocol (6694 ± 178 *Vs *8160 ± 184 Kcal, -18%) was shown, no significant differences were observed (Fig 1). Thus, animals were pair-fed for global energy intake. Lipid energy of L rats was almost four times higher than S rats (2577 ± 68 *vs *628 ± 72 Kcal), which was counterbalanced by lower glucid (2778 ± 66 *vs *5891 ± 62 Kcal) and, in a smaller proportion, of protein (1339 ± 53 *vs *1632 ± 45 Kcal) energy.

High fat diet rats had lower body mass (431 ± 38 *Vs *468 ± 25 g, p = 0.003), lean body mass (369 ± 18 *vs *409 ± 23 g, p = 0.0006); there was no significant differences for the left perirenal fat pads (6.0 ± 1.8 *Vs *5.5 ± 1.1, p = 0.3) – Table 1. Bone parameters BMC, BMD and SA in L rats diet rats were lower than in S rats: (11 ± 0.5 *Vs *13 ± 0.7 g, p = 0.0004, 0.158 ± 0.006 *Vs *0.167 ± 0.004 g/cm^2^, p = 0.0009, 72 ± 3 *Vs *76 ± 4 cm^2^, p = 0.03) – Table 2. Significant correlations were observed between body composition and bone parameters (Table 3).

**Table 1:** Body composition in S and L rats (means ± SD of LBM : lean body mass, FM : fat mass, PFP : peri renal fat pad)

**Groups**	**Body mass (g)**	**LBM (g)**	**FM (g)**	**PFP (g)**
**S**	468 ± 25	409 ± 23	80 ± 17	5.5 ± 1.1
**L**	431 ± 38	369 ± 18	82.5 ± 17	6.0 ± 1.8
Difference	-8%	-10%	+3%	+ 4.5%
p	0.003	0.0006	0.7	0.3

**Table 2:** Bone parameters in S and L rats (means ± SD).

**Groups**	**BMC(g)**	**Skeleton area (cm^2^)**	**BMD(g/cm^2^)**
**S**	13 ± 0.7	76 ± 4	0.167 ± 0.004
**L**	11 ± 0.5	72 ± 3	0.158 ± 0.006
Difference	-15%	-5.3%	-5.4%
p	0.0004	0.03	0.0009

**Table 3:** Correlations between body composition, adiponectin and bone parameters.

	**Body mass (g)**	**LBM (g)**	**PFP (G)**	**Adiponectin (μg/ml)**
**BMC (g)**	r = 0.73 p = 0.0002	r = 0.78 p = 0.0001	ns	Ns
**SA (cm^2^)**	r = 0.62 p = 0.003	r = 0.72 p = 0.0002	ns	Ns
**BMD (g/cm^2^)**	r = 0.50 p = 0.03	r = 0.44 p = 0.05	r = -0.50 p = 0.02	r = -0.62 p = 0.03

Body mass is correlated with SA (r = 0.62, p = 0.003), BMC (r = 0.73, p = 0.0002), BMD (r = 0.50, p = 0.03); LBM is correlated with SA (r = 0.72, p = 0.0002), BMC (r = 0.78, p < 0.0001) and to BMD (r = 0.44, p = 0.05); Peri renal fat pad is negatively correlated with BMD (r = -050, p = 0.02). There were no significant correlations between bone parameters and total fat mass. Adiponectin mean level is lower in L than in S rats and individual values are negatively correlated with BMD (r = -0.62, p = 0.03).

## Discussion

The aim of this study was to investigate changes of some bone parameters in growing rats fed with a high fat diet and also to evaluate possible correlations between body composition and bone parameters. All bone parameters (BMC, BMD, SA) measured were lowered by the high fat diet. Another salient feature of this study is the negative correlation noted between visceral fat and BMD and the positive correlation between adiponectin and BMD.

Bone characteristics are represented by 2 factors: the volumetric development which is reflected by the skeleton area (SA) and the calcium accretion reflected by the BMD. The product of these 2 parameters is the BMC given by DXA. The first factor depends mainly on the protein synthesis needed to build the bone structure. This statement is sustained by the significant high correlation (p = 0.0002) found between SA and LBM. Indeed, protein synthesis must be related to protein intake and also energy intake, especially glucidic, needed for the synthesis. As shown before, these two parameters are lower in L than in S rats. We have already discussed this point for LBM in a previous paper [11]. For bone, other studies have pointed the major role of a correct glucido-protidic intake for an optimal development [5,12-14]. The second factor (BMD) depends on calcium intake, absorption and accretion. With respect to calcium intake, L rats consumption was not very different from S: 0.8 g/100 g vs 0.9 g/100 g food. Considering a mean intake per day of 20 g food, this corresponds to 160 mg/day of calcium per rat. The absorption has not been determined in this survey, but it may be stated that it was lowered considering the high fat level in the diet, as underlined in previous studies [4,15,16]. The D3 vitamin, which facilitates calcium absorption was probably more concentrated in L food than in S, since the oil added (D3 enriched) brought a supplement of this liposoluble vitamin. Whatever the calcium inbalance, its accretion was lowered in L rats, as attested by the lower BMD, and the correlation between the visceral fat and the BMD must be underlined. Some previous studies have evoked the negative role on bone of an unbalanced diet, very rich in fat [2-5,7] and more specifically of unbalanced fat composition, especially rich in palmitoleic acid (saturated) fat [6]. The studies on the relationships betwen body composition and bone have mainly focused on the correlations between fat mass (a component of body mass) and BMD. Most of these studies conducted in elderly people reported a BMD increase of bearing bones in overweight people with significant correlations between BMD and the two components of body mass, the LBM and also the FM [17,18]. In young women, there was not a real consensus with respect to these relationships [19,20]. In the present study, we reported a high correlation between body mass and BMD, but no significant correlations merged between fat mass and the bone parameters. In contrast, we observed a negative correlation between visceral fat mass and BMD but not with skeletal area (SA), thus a specific action of this visceral fat on the calcium deposition. This observation conflicted with a previous study reporting a positive correlation between visceral fat (estimated from waist circumference) and BMD in post-menopausal women [8]. Some evidence suggested that fat mass acts on BMD not only via mechanical factors, but also by means of adipokines like leptin and adiponectin. A negative correlation between adiponectin and BMD was reported [9]. Similarly, we noted a negative correlation between adiponectin and BMD in these growing rats.

It may be concluded that there are some close correlations between fat and BMD. More specifically, the visceral fat, which was already pointed out as a reliable marker of insulin resistance, in humans [21,22] seems to present the same deleterious effects on bone. This study confirms in growing rats that a high fat diet is pathogenic, including bone metabolism, but rather than considering diet composition, it is suggested that all kind of diets and/or life-style inducing an excess amount of visceral fat is deleterious for bone.
